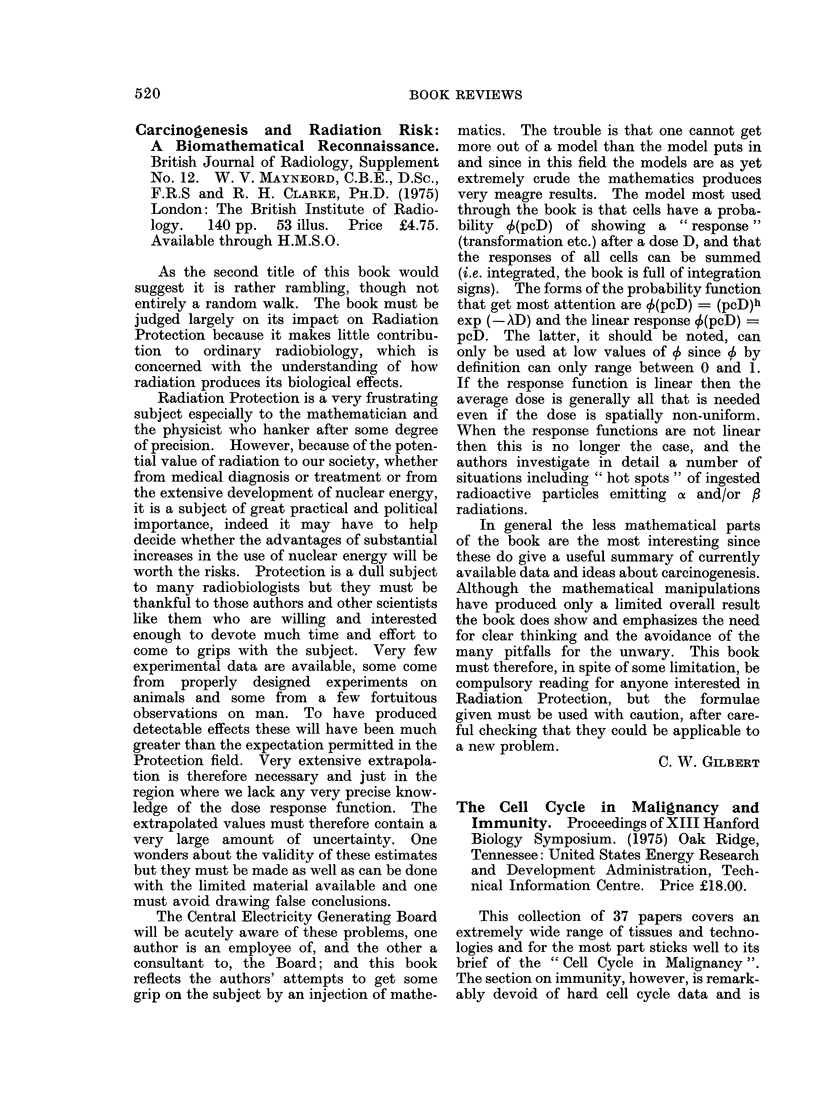# Carcinogenesis and Radiation Risk: A Biomathematical Reconnaissance

**Published:** 1975-10

**Authors:** C. W. Gilbert


**Carcinogenesis and Radiation Risk: A Biomathematical Reconnaissance.** British Journal of Radiology, Supplement No. 12. W. V. Mayneord, C.B.E., D.Sc., F.R.S and R. H. Clarke, Ph.D. (1975) London: The British Institute of Radiology. 140 pp. 53 illus. Price £4.75. Available through H.M.S.O.

As the second title of this book would suggest it is rather rambling, though not entirely a random walk. The book must be judged largely on its impact on Radiation Protection because it makes little contribution to ordinary radiobiology, which is concerned with the understanding of how radiation produces its biological effects.

Radiation Protection is a very frustrating subject especially to the mathematician and the physicist who hanker after some degree of precision. However, because of the potential value of radiation to our society, whether from medical diagnosis or treatment or from the extensive development of nuclear energy, it is a subject of great practical and political importance, indeed it may have to help decide whether the advantages of substantial increases in the use of nuclear energy will be worth the risks. Protection is a dull subject to many radiobiologists but they must be thankful to those authors and other scientists like them who are willing and interested enough to devote much time and effort to come to grips with the subject. Very few experimental data are available, some come from properly designed experiments on animals and some from a few fortuitous observations on man. To have produced detectable effects these will have been much greater than the expectation permitted in the Protection field. Very extensive extrapolation is therefore necessary and just in the region where we lack any very precise knowledge of the dose response function. The extrapolated values must therefore contain a very large amount of uncertainty. One wonders about the validity of these estimates but they must be made as well as can be done with the limited material available and one must avoid drawing false conclusions.

The Central Electricity Generating Board will be acutely aware of these problems, one author is an employee of, and the other a consultant to, the Board; and this book reflects the authors' attempts to get some grip on the subject by an injection of mathematics. The trouble is that one cannot get more out of a model than the model puts in and since in this field the models are as yet extremely crude the mathematics produces very meagre results. The model most used through the book is that cells have a probability $\phi(\text{pcD})$ of showing a "response" (transformation etc.) after a dose D, and that the responses of all cells can be summed (*i.e.* integrated, the book is full of integration signs). The forms of the probability function that get most attention are $\phi(\text{pcD}) = (\text{pcD})^{h} \exp(-\lambda D)$ and the linear response $\phi(\text{pcD}) = \text{pcD}$. The latter, it should be noted, can only be used at low values of $\phi$ since $\phi$ by definition can only range between 0 and 1. If the response function is linear then the average dose is generally all that is needed even if the dose is spatially non-uniform. When the response functions are not linear then this is no longer the case, and the authors investigate in detail a number of situations including "hot spots" of ingested radioactive particles emitting $\alpha$ and/or $\beta$ radiations.

In general the less mathematical parts of the book are the most interesting since these do give a useful summary of currently available data and ideas about carcinogenesis. Although the mathematical manipulations have produced only a limited overall result the book does show and emphasizes the need for clear thinking and the avoidance of the many pitfalls for the unwary. This book must therefore, in spite of some limitation, be compulsory reading for anyone interested in Radiation Protection, but the formulae given must be used with caution, after careful checking that they could be applicable to a new problem.

C. W. Gilbert